# Revealing the potent probiotic properties and alcohol degradation capabilities of *Lactiplantibacillus plantarum* BGI-J9 by combining complete genomic and phenotypic analysis

**DOI:** 10.3389/fmicb.2025.1664033

**Published:** 2025-09-09

**Authors:** Yaqi Fan, Zhihui Ma, Benliang Wei, Yangfeng Wen, Xiaowei Xu, Xing Rao, Zhinan Wu, Yanhong Liu, Haifeng Zhang, Yiyi Zhong, Yuanqiang Zou, Liang Xiao

**Affiliations:** ^1^BGI Research, Shenzhen, China; ^2^College of Life Sciences and Oceanography, Shenzhen University, Shenzhen, China; ^3^BGI Precision Nutrition (Shenzhen) Technology Co., Ltd, Shenzhen, China; ^4^College of Plant Protection, Hunan Agricultural University, Changsha, China; ^5^College of Life Sciences, Southwest University, Chongqing, China; ^6^College of Life Sciences, University of Chinese Academy of Sciences, Beijing, China; ^7^Shenzhen Engineering Laboratory of Detection and Intervention of Human Intestinal Microbiome, BGI Research, Shenzhen, China; ^8^State Key Laboratory of Genome and Multi-omics Technologies, BGI Research, Shenzhen, China

**Keywords:** *Lactiplantibacillus plantarum*, complete genome, antioxidation, alcohol dehydrogenase, acetaldehyde dehydrogenase, ethanol metabolism

## Abstract

Probiotics have demonstrated broad prospects in maintaining human health, and complete genome analysis enables unveiling the intrinsic probiotic mechanisms. In this study, the probiotic properties of *Lactiplantibacillus plantarum* BGI-J9 (BGI-J9) were explored via integrating complete genomic and phenotypic analysis. Results indicated that the high-quality complete genome of BGI-J9 comprises 3,128,867 bp with 2,926 coding sequences and an average GC content of 45%. Genomic annotation analysis revealed that BGI-J9 harbored *nhaK*, *plsC*, *pyk*, *atp*, *opp*, *rps*, *rpl*, *rpm* system genes, as well as plantaricin, glutathione peroxidase family, glutathione, catalase, thioredoxin encoding genes, and exhibited favorable gastrointestinal tolerance, antimicrobial activity, antioxidant activity in *in vitro* assays. Notably, alcohol degradation enzyme genes were identified in the BGI-J9 genome, which accounted for the potent *in vitro* alcohol dehydrogenase and acetaldehyde dehydrogenase activities and alcohol degradation capacity exhibited by BGI-J9. These findings indicated that BGI-J9 has the potential to assist in promoting alcohol degradation and mitigating alcohol-induced damage. In conclusion, this study first presented the complete genome of BGI-J9, furnishing a theoretical basis for its application in alleviating alcohol damage.

## 1 Introduction

According to the guidelines of the International Agency for Research on Cancer (IARC), alcohol is classified as a Group 1 human carcinogen, indicating a clear link to the development of human cancers (Gao et al., 2024; Ratna and Mandrekar, 2017). Alcohol exerts multifaceted detrimental effects on the human body. Excessive alcohol metabolism could increase hepatic oxygen consumption and generate excess reactive oxygen species (ROS), inducing oxidative stress and inflammatory reactions to damage the liver (Shim and Jeong, 2020). Additionally, ROS activated inflammatory pathways, triggering inflammatory responses that further contribute to liver injury (Shim and Jeong, 2020). Besides, acetaldehyde, a toxic intermediate product during alcohol metabolism, could form adducts with cellular DNA and proteins, exacerbate oxidative stress, thereby impairing hepatocyte function (Setshedi et al., 2010; Vogt and Richie, 2007). What’s worse, Chronic alcohol consumption could cause gut microbiota disorder, which could compromise the intestinal mucosal barrier and induce inflammation, ultimately leading to alcoholic liver disease and cirrhosis (Hao et al., 2023; Kawaratani et al., 2013). While some commercially available anti-hangover medications offer some calming and soothing effects, they are unable to diminish the damage caused by alcohol and also have several side effects (Perrin et al., 2024). Reducing alcohol intake is key to healthy living, but scoping out novel means of mitigating alcohol damage is more in line with consumer demands.

Recently, the intensive connection between gut microbiota disorders and liver injury has been emphasized continuously. The bi-directional interaction between the gut and the liver, the “gut-liver” axis, has emerged as a pivotal point in liver disease investigations (Tilg et al., 2022). As essential components of the gut microbiota, probiotics have demonstrated considerable potential in ameliorating alcoholic liver disease. *Lactiplantibacillus plantarum* (*L. plantarum*) is a representative species of lactobacili, with numerous strains confirmed to possess probiotic functions. These strains exhibit multiple probiotic properties, including auto-aggregation, cell surface hydrophobicity, hydrogen peroxide production, cellular adhesion, modulation of metabolism and reduction of inflammation (Stojanov et al., 2024; Jeong et al., 2021). Research indicated that selected probiotic strains, particularly within *L. plantarum*, confer hepatoprotective effects against alcohol-induced injury through multiple mechanisms. Primary mechanisms include: mitigates alcohol-induced gut microbiota dysbiosis and inflammatory responses by modulating microbial composition, suppressing pro-inflammatory factor production and enhancing anti-inflammatory factor generation (Sakurai et al., 2022), attenuates host ethanol absorption by enhancing the activities of alcohol dehydrogenase (ADH) and aldehyde dehydrogenase (ALDH) (Zhang et al., 2024) or enhancing hepatic antioxidant capacity via elevated levels of superoxide dismutase (SOD) and glutathione (GSH), thereby mitigating inflammatory and oxidative injury pathways (Gan et al., 2021; Wang T. et al., 2023). Heterologous expression of the ALDH gene from *L. plantarum* enables the synthesis of metabolic enzymes involved in alcohol degradation, thereby directly participating in the ethanol metabolic pathway (Wang Z. et al., 2023). These studies primarily focused on the effects of probiotic intervention in improving alcohol-induced damage. However, few reports are available on how to efficiently screen probiotics that promote alcohol metabolism and alleviate damage. Advances in high-throughput sequencing technology has enabled researchers to obtain high-quality, complete bacterial genomes. This facilitates efficient genomic-level screening of probiotics, revealing strains with the potential to improve alcohol metabolism through gene mining. Combining genomic mining with *in vitro* experiments helps analyze probiotic functions in deeper levels, obtain comprehensive and convincing probiotic evaluation data, and explore their novel application potential.

*Lactiplantibacillus plantarum* BGI-J9 (BGI-J9) was a strain previously isolated from Inner Mongolia traditional fermented yogurt, which exhibited promising *in vitro* probiotic properties. However, further investigations are required for the application of BGI-J9 in food and medicine. The purpose of this study is to comprehensively analyze the genome of BGI-J9 by complete genome sequencing, along with phenotypic analysis to reveal the intrinsic factors of BGI-J9 for health benefits, especially in accelerating alcohol metabolism.

## 2 Materials and methods

### 2.1 Materials and cultural conditions of bacteria

BGI-J9 was isolated from an Inner Mongolia traditional fermented yogurt. BGI-J9 and *Lactiplantibacillus plantarum* 299v (299v) were cultured in MRS medium (Hope Bio-Technology, Qingdao, China) at 37 °C for 24 h. Five pathogenic strains, including *Staphylococcus aureus* (*S. aureus*) ATCC 29213, *Escherichia coli* (*E. coli*) ATCC 25922, *Pseudomonas aeruginosa* (*P. aeruginosa*) ATCC 9027, *Fusobacterium nucleatum* (*F. nucleatum*) ATCC 25586 and *Enterobacter cloacae* (*E. cloacae*) ATCC 23355 were cultured in BHI medium (Aobox Biotechnology, Beijing, China) at 37 °C for 24 h, *F. nucleatum* ATCC 25586 need to be cultured in an anaerobic environment (80% N_2_, 15% CO_2_, 5% H_2_) under the above conditions. All the strains were deposited in Shenzhen Engineering Laboratory of Detection and Intervention of human intestinal microbiome.

### 2.2 Morphological analysis

The morphology of bacteria was analyzed by staining using a Gram staining kit (Sangon Biotech, Shanghai, China). The bacteria were stained according to the manufacturer’s instructions and were observed under an optical microscope (1000×). Then, the bacteria cells were fixed at 4 °C with 2.5% glutaraldehyde for 4 h and dehydrated with gradient concentration ethanol. The obtained samples were observed and photographed under the electron microscope (Hitachi SU8100, Hitachi Ltd., Tokyo, Japan).

### 2.3 Complete genome extraction, sequencing, and assembly of BGI-J9

BGI-J9 was cultured overnight and harvested by centrifugation at 10,000 × g for 10 min. The bacterial cells were washed three times with sterile physiological saline to obtain a pure bacterial pellet. Genomic DNA was extracted using a Rapid Bacterial Genomic DNA Isolation Kit (Sangon Biotech, Shanghai, China).

For sequencing, short-read data were generated using the DNBSEQ-T5 platform (BGI, Shenzhen, China), while long-read data were obtained using the CycloneSEQ-WT02 platform (BGI, Shenzhen, China) (Liang et al., 2025). Subsequently, quality control was performed. Short-read sequences shorter than 90 base pairs (bp) or containing more than three ambiguous bases were filtered out using fastp v0.23.4. Adapter sequences (AAGTCGGAGGCCAAGCGGTCTTAGGAAGACAA and AAGTCGGATCGTAGCCATGTCGTCGTTCTGTGAGCCAAGGAGTTG) were identified, and Q20 > 97.5% was applied. For long-read sequences, reads with a quality score below 10 or shorter than 1,000 bp were filtered out using NanoFilt v2.8.0.

Hybrid assembly of the short-read and long-read sequencing data were performed using Unicycler v0.4.8, resulting in a complete genome sequence. The quality of the assembled genome was assessed using CheckM v1.0.2.

### 2.4 Genome annotation and analysis

In total, the genomes of 32 bacterial strains (26 strains of *L. plantarum*, five representative strains of *Lacticaseibacillus, Limosilactobacillus*, *Lactobacillus* species, and one strain of *Streptococcus salivarius*) were downloaded from NCBI and compared with the genome of BGI-J9 for phylogenetic analysis. Taxonomic annotation and phylogenetic analysis were conducted based on the Genome Taxonomy Database Toolkit (GTDB-Tk). The resulting evolutionary tree was visualized and annotated using the Interactive Tree Of Life (iTOL) online platform.^1^ Average Nucleotide Identity (ANI) analysis was performed by JSpeciesWS.^2^

Genome annotation was performed using Prokka v1.14.6, and circular genome visualization was generated using Proksee.^3^ The complete genome was categorized into COG (Clusters of Orthologous Groups) classifications using the EggNOG database.^4^ Functional annotation of genes from the BGI-J9 genome was conducted using the KEGG database.^5^ Potential bacteriocin gene clusters in BGI-J9 were predicted using BAGEL4.^6^ The secondary metabolite biosynthetic gene clusters were predicted by antiSMASH version 8.0.0.^7^

### 2.5 Digestive tract environment tolerance test

Following the method described by Wang et al. (2025), the survival rates of strain BGI-J9 following 2 h of incubation at 37 °C were evaluated in simulated gastric fluid (pH 2 and pH 3), simulated intestinal fluid and 0.3% bile salts. The reagents used were all purchased from Thermo Fisher Scientific, USA and Yuanye Bio-Technology, Shanghai, China.

### 2.6 Antibacterial activity test

Antimicrobial activity of strain BGI-J9 against five pathogenic bacteria (including *S. aureus* ATCC 29213, *E. coli* ATCC 25922, *P. aeruginosa* ATCC 9027, *E. cloacae* ATCC 23355 and *F. nucleatum* ATCC 25586) were evaluated according to the method described by Wang et al. (2025).

### 2.7 The antioxidant activity measurement

The radicals (including 1,1-Diphenyl-2-picrylhydrazyl radical (DPPH), 2,2′-Azino-bis (3-ethylbenzothiazoline-6-sulfonic acid) radical cation (ABTS) and hydroxyl radical) scavenging capacity were tested according to previous studies (Foti, 2015; Kut et al., 2023; Treml and Šmejkal, 2016) using commercial regents (Sangon Biotech and Macklin Biochemical Technology, Shanghai, China).

### 2.8 *In vitro* alcohol degrading enzyme activity quantification

The activated bacterial pellets of BGI-J9 and 299v were centrifuged (10,000 rpm, 10 min), collected and washed twice with distilled water. Then, 200 μL of cell lysis buffer (0.05 mol/L) and 20 μL of lysozyme solution (20 mg/mL) were added to the pellet, followed by the addition of *β*-mercaptoethanol to a final concentration of 5 mmol/L. The mixture was incubated at 37 °C for 60 min. After incubation, the sample was frozen at −20 °C for 5 min and then centrifuged (10,000 rpm, 15 min) to collect the pellet. The pellet was resuspended in PBS buffer (pH 8.8) at a solid-to-liquid ratio of 1:5, followed by centrifugation (10,000 rpm, 20 min). The supernatant was collected as the crude extract of ADH and ALDH.

The activities of ADH and ALDH were measured using the ADH Assay Kit and ALDH Assay Kit (both purchased from Sangon Biotech, Shanghai, China), following the manufacturer’s instructions.

### 2.9 *In vitro* alcohol degradation assay

Overnight cultured BGI-J9 and 299v were prepared as experimental samples. An equal volume (1%, v/v) of the test samples was added into the MRS broth with 5% (v/v) ethanol and incubated at 37 °C for 24 h. The ethanol concentration in the MRS broth was measured at 0, 6, 12, 18 and 24 h using an ethanol content detection kit (Solarbio, Beijing, China). MRS broth with 5% (v/v) ethanol but without bacteria inoculation was used as a control to account for ethanol evaporation.

### 2.10 Data analysis methods

All *in vitro* experiments were performed in triplicate, with data presented as mean ± standard deviation (SD). Statistical analysis was conducted using SPSS v27 (IBM, USA), including Student’s t-test and one-way ANOVA. Statistical significance threshold of *p* < 0.05. Data visualization was performed using GraphPad Prism v9.5 (GraphPad Software, USA).

## 3 Results

### 3.1 Morphological characteristics of BGI-J9

**Figure 1:**
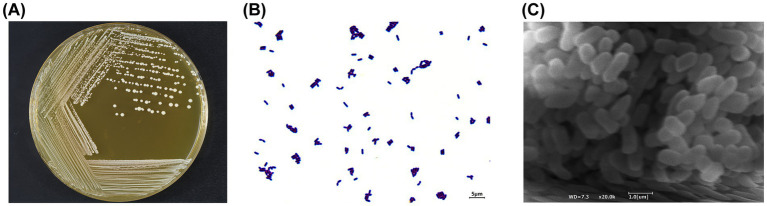
Morphological characteristics of BGI-J9. **(A)** The colony morphology of BGI-J9; **(B)** cell morphology under Gram staining (1000×); **(C)** cell morphology (20,000 ×) under an electron microscope.

After incubation on MRS solid medium at 37 °C for 24 h, BGI-J9 formed raised and circular colonies with a milky-white appearance and smooth margins (Figure 1A). As shown in Figure 1B, Gram staining results showed that BGI-J9 cells were stained purple and rod-shaped, indicating that BGI-J9 is a Gram-positive bacterium. After being magnified 20,000 times, the cells of BGI-J9 exhibited a typical bacilli shape, mostly in the form of short rods (Figure 1C).

### 3.2 General genomic features

**Figure 2:**
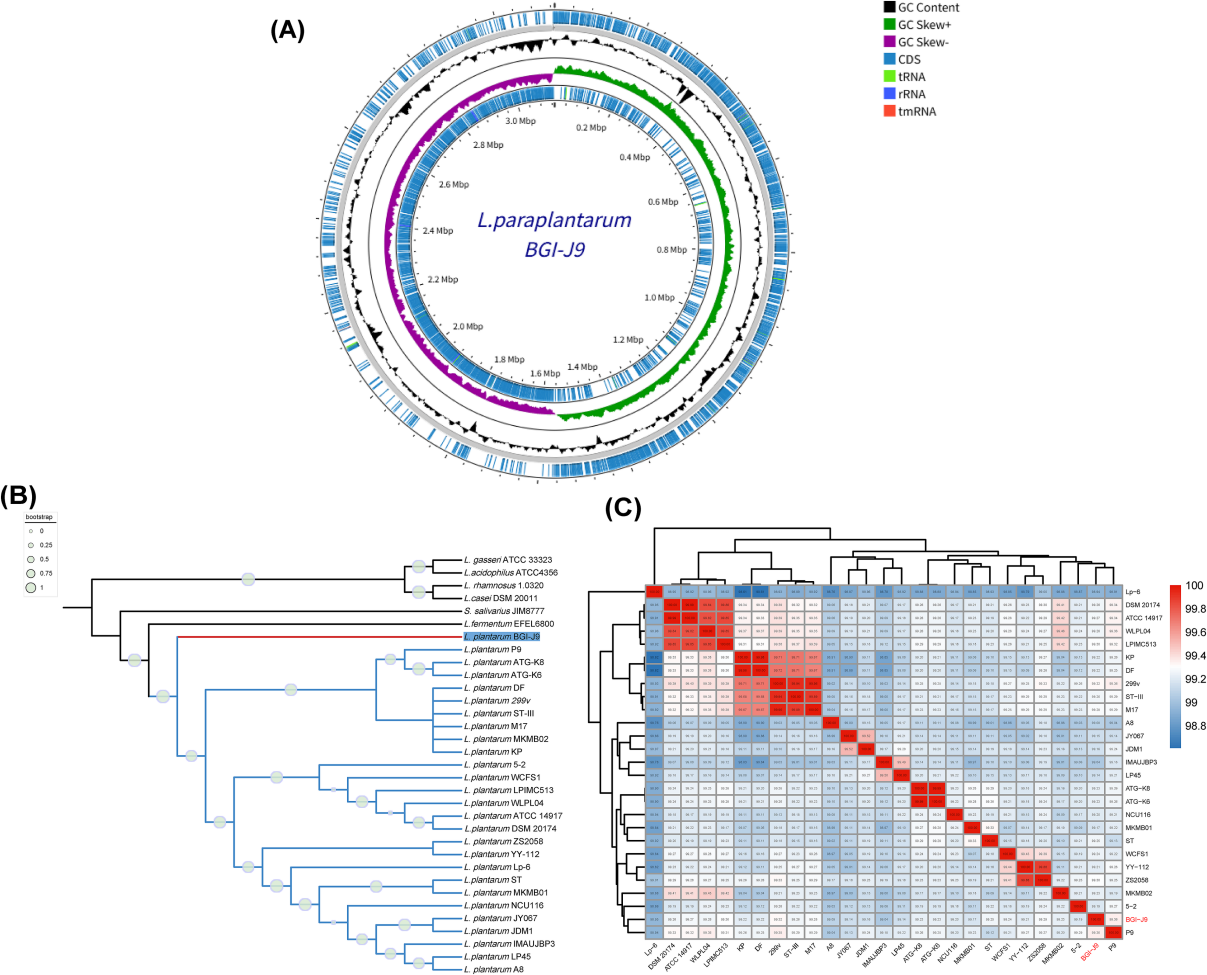
Genomic features of BGI-J9. **(A)** Circular genome map of BGI-J9; **(B)** phylogenetic relationship between BGI-J9 and reference strains; **(C)** ANI heatmap of *L. plantarum* strains.

High completeness (100%) and low contamination circle genome map of BGI-J9 displayed in Figure 2A, which consists of two contigs with a total length of 3,128,867 bp. Genomic analysis showed a GC content of 45% and annotated 2,926 protein coding sequences, along with 16 rRNA, 70 tRNA, and one tmRNA genes. Interestingly, phylogenetic analysis indicated that BGI-J9 clustered within a distinct clade while maintaining close phylogenetic affinity with other *L. plantarum* strains (Figure 2B). This specific branching pattern suggested that BGI-J9 might represent a unique evolutionary lineage within *L. plantarum*, potentially harboring distinctive biological characteristics that differentiate it from other strains during evolutionary divergence. The phylogenetic tree exhibited high reliability, as indicated by bootstrap values approaching 1 for the BGI-J9-containing clade. Subsequent ANI analysis (Figure 2C) demonstrated that BGI-J9 showed the highest genomic similarity with strains *L. plantarum* P9 (ANI = 99.36%) and 299v (ANI = 99.32%), confirming their closest evolutionary relationship and potentially sharing similar probiotic function.

### 3.3 Functional annotation of BGI-J9 genome

**Figure 3:**
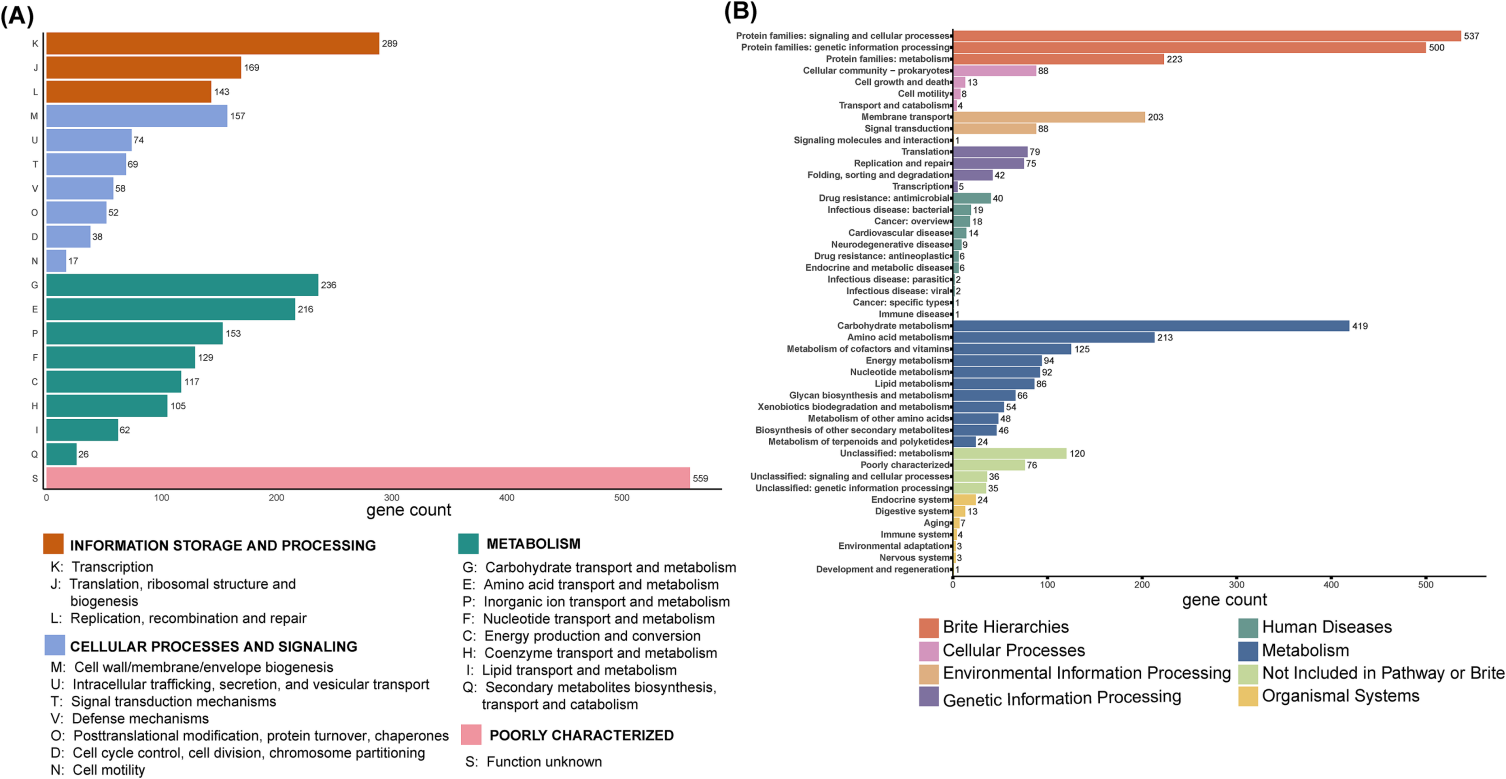
Functional annotation of BGI-J9. Function annotation of genes from BGI-J9 based on **(A)** the COG database and **(B)** KEGG database.

As shown in Figure 3A, functional annotation results indicated that a total of 2,669 proteins were annotated in the COG database and classified into 19 functional categories across 4 major classes: information storage and processing (601 proteins), cellular processes and signaling (465 proteins), metabolism (1,044 proteins), poorly characterized (559 proteins). In the KEGG database, 3,573 genes from BGI-J9 were annotated to 8 primary pathways, including 1,267 genes in metabolic pathways, 1,260 in protein family-related pathways, 292 in environmental information processing pathways, 201 in genetic information processing pathways, 118 in human disease-related pathways, 113 in cellular processes pathways, 55 in human organ system-related pathways, these genes support the growth, metabolism, and function of BGI-J9 (Figure 3B). Notably, carbohydrate metabolism (419 genes) accounted for the highest proportion of metabolic pathways, followed by amino acid metabolism (216 genes), metabolism of cofactors and vitamins (125 genes). Further CAZyme database annotation revealed that BGI-J9 possesses 27 glycosyltransferase genes, 22 glycoside hydrolase genes, along with genes encoding auxiliary activity redox enzymes and carbohydrate-binding modules (Supplementary Table S1). Abundant synthesis and metabolism of carbohydrates and metabolites genes in BGI-J9 not only endowed it with the robust growth capacity but also the potential for metabolic and functional diversity.

### 3.4 Tolerance of BGI-J9 in digestive tract environments

**Figure 4:**
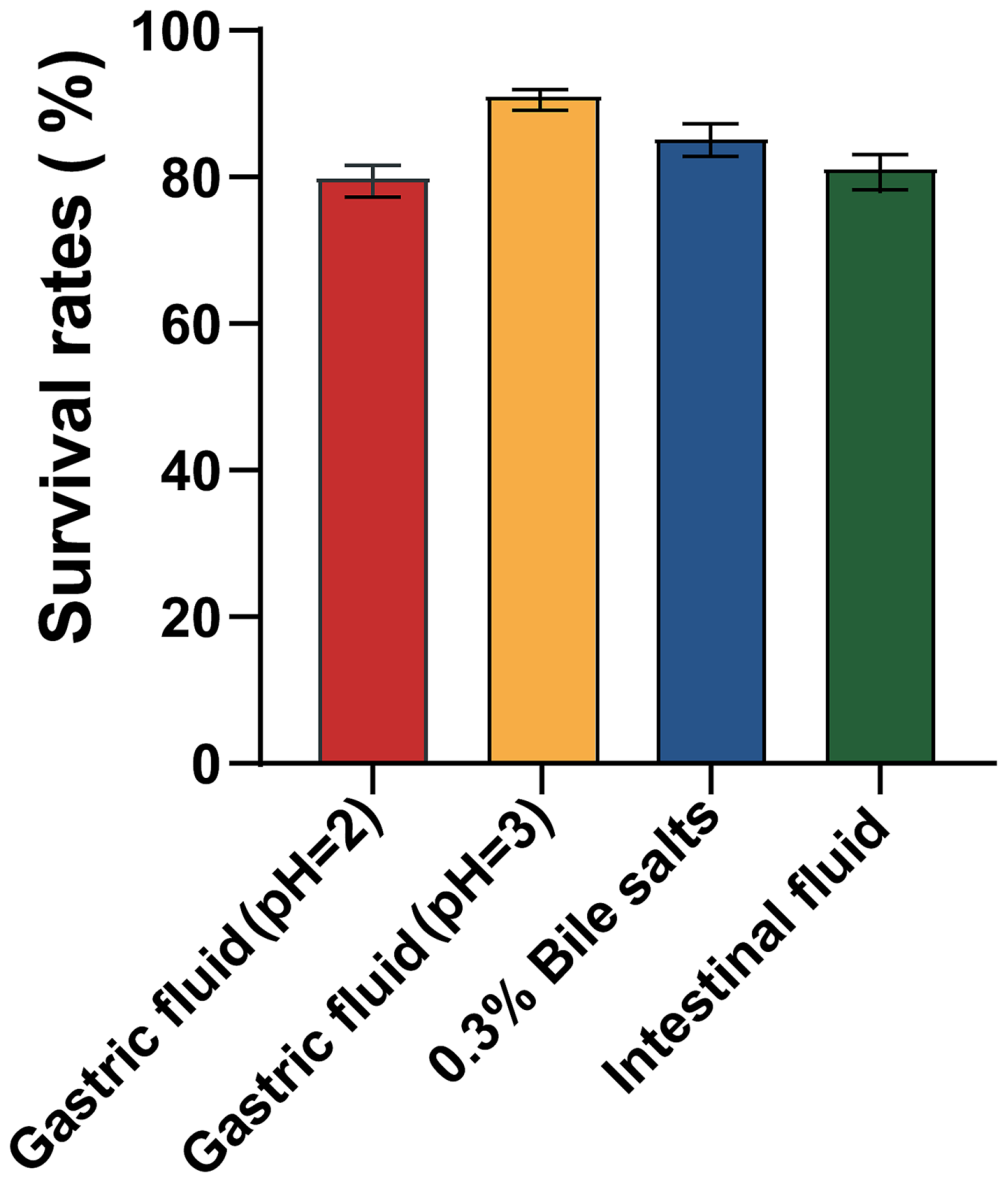
Survival rates of BGI-J9 in simulated gastrointestinal digestive fluids.

Tolerance to extreme gastrointestinal environments is the foundation for probiotic to colonize and exert function. Genome annotation of BGI-J9 revealed that 21 and 36 genes associated with acid and bile salt tolerance were identified in the genome of BGI-J9, respectively (Table 1). BGI-J9 resists pH damage mainly through the synergistic effects of multiple proteins, including pH regulation system proteins (*plsC*), Na^+^/H^+^ antiporters (*nhaK*) and proton pump subunits (*atpA-atpH*). Additionally, bile salt tolerance genes were identified in BGI-J9, operating through hydrolyze bile salts (*cbh*), membrane stabilization (*cfa*), nutrient transport (*opp* system), ribosomal proteins (*rps, rpl, rpm*) and metabolic enzymes (*ppaC, pepO, lpd, glnA*). The genome annotation also identified several genes associated with energy metabolism (*celA, celB, celC*) and digestive enzyme resistance genes (Supplementary Table S2) that promote gut persistence. The aforementioned genes collectively confer the potential for BGI-J9 to resist extreme stresses in the gastrointestinal tract. These were confirmed by the *in vitro* assay results displayed in Figure 4 that BGI-J9 demonstrated survival rates exceeding 80% in gastric fluid, bile salts and intestinal fluid. These results indicated that BGI-J9 could withstand extreme gastrointestinal conditions, thereby enhancing the potential for colonization in the intestinal tract.

**Table 1:** Gastrointestinal environment tolerance-related genes in BGI-J9.

Tolerance Items	KO number	Genes	Description	Gene count
Acid	K02111	*atpA*	F-type H^+^/Na^+^-transporting ATPase subunit alpha	1
Acid	K02108	*atpB*	F-type H^+^-transporting ATPase subunit a	1
Acid	K02114	*atpC*	F-type H^+^-transporting ATPase subunit epsilon	1
Acid	K02112	*atpD*	F-type H^+^/Na + −transporting ATPase subunit beta	1
Acid	K02110	*atpE*	F-type H^+^-transporting ATPase subunit c	1
Acid	K02109	*atpF*	F-type H^+^-transporting ATPase subunit b	1
Acid	K02115	*atpG*	F-type H^+^-transporting ATPase subunit gamma	1
Acid	K02113	*atpH*	F-type H^+^-transporting ATPase subunit delta	1
Acid	K03316	*nhaK*	Sodium proton antiporter	4
Acid	K00655	*plsC*	1-acyl-sn-glycerol-3-phosphate acyltransferase	1
Acid	K01580	*gadB*	glutamate decarboxylase	7
Acid	K00873	*pyk*	Pyruvate kinase	1
Bile	K01442	*cbh*	Cholylglycine hydrolase	3
Bile	K00574	*cfa*	Cyclopropane-fatty-acyl-Phospholipid synthase	2
Bile	K01915	*glnA*	Glutamine synthetase	1
Bile	K00382	*lpd*	Dihydrolipoyl dehydrogenase	1
Bile	K15580	*oppA*	Oligopeptide transport system substrate-binding protein	9
Bile	K15581	*oppB*	Oligopeptide transport system permease protein	1
Bile	K15582	*oppC*	Oligopeptide transport system permease protein	1
Bile	K15583	*oppD*	Oligopeptide transport system ATP-binding protein	1
Bile	K10823	*oppF*	Oligopeptide transport system ATP-binding protein	1
Bile	K07386	*pepO*	Putative endopeptidase	1
Bile	K15986	*ppaC*	Manganese-dependent inorganic pyrophosphatase	1
Bile	K02939	*rplL*	Large subunit ribosomal protein L9	1
Bile	K02884	*rplS*	Large subunit ribosomal protein L19	1
Bile	K02887	*rplT*	Large subunit ribosomal protein L20	1
Bile	K02888	*rplU*	Large subunit ribosomal protein L21	1
Bile	K02899	*rpmA*	Large subunit ribosomal protein L27	1
Bile	K02902	*rpmB*	Large subunit ribosomal protein L28	1
Bile	K02916	*rpmI*	Large subunit ribosomal protein L35	1
Bile	K02945	*rpsA*	Small subunit ribosomal protein S1	1
Bile	K02967	*rpsB*	Small subunit ribosomal protein S2	1
Bile	K02986	*rpsD*	Small subunit ribosomal protein S4	1
Bile	K02956	*rpsO*	Small subunit ribosomal protein S15	1
Bile	K02959	*rpsP*	Small subunit ribosomal protein S16	1
Bile	K02968	*rpsT*	Small subunit ribosomal protein S20	1
Bile	K02970	*rpsU*	Small subunit ribosomal protein S21	1
Gut persistence	K02760	*celA*	Cellobiose PTS system EIIB component	2
Gut persistence	K02761	*celB*	Cellobiose PTS system EIIC component	8
Gut persistence	K02759	*celC*	PTS system, Lactose/Cellobiose specific IIA subunit	4

### 3.5 Antimicrobial activity of BGI-J9

**Figure 5:**
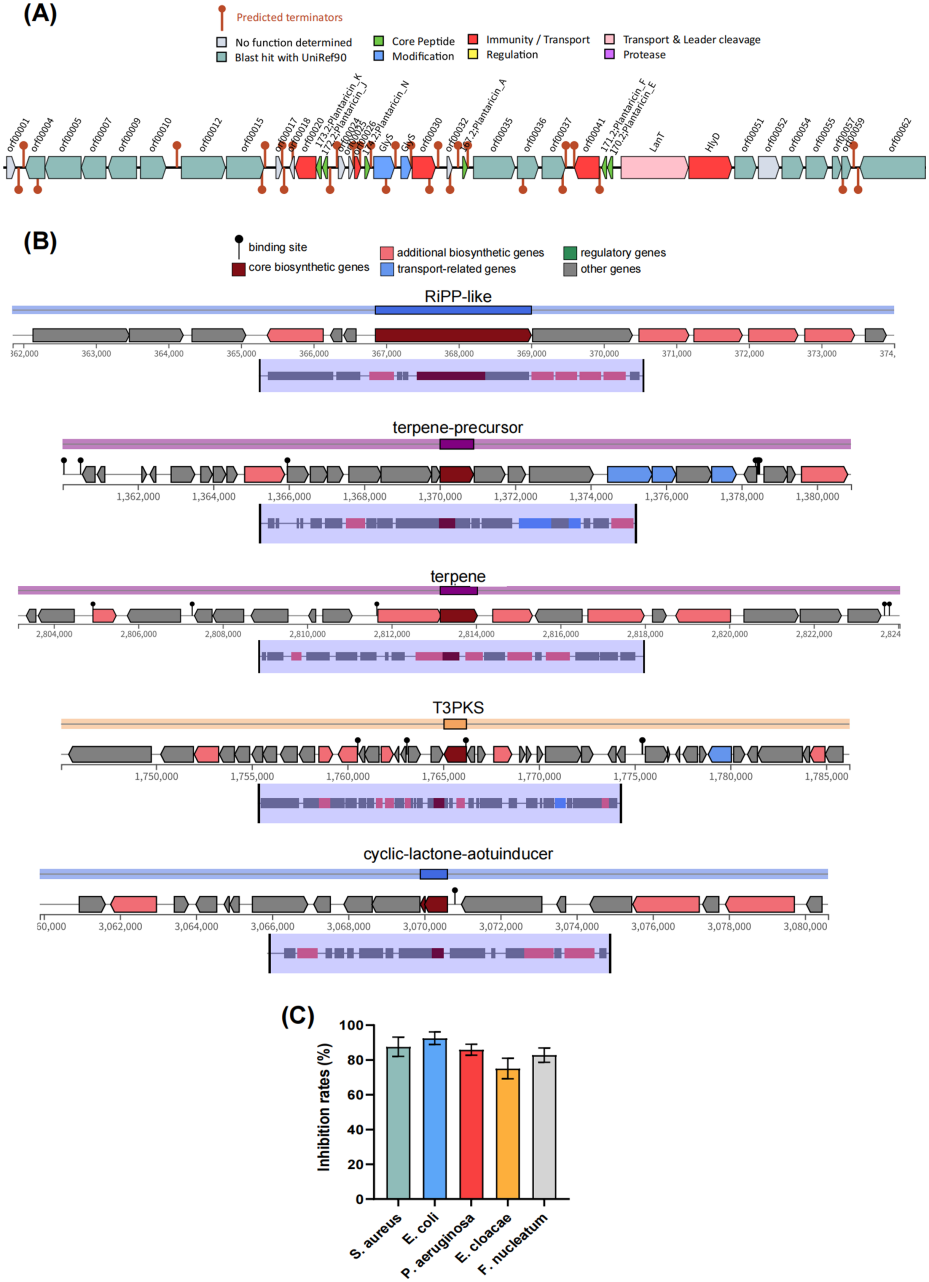
Antimicrobial activity analysis of BGI-J9. **(A)** The predicted bacteriocin gene cluster in BGI-J9; **(B)** antimicrobial activity of BGI-J9 against five pathogens; **(C)** secondary metabolite biosynthetic gene clusters in BGI-J9.

Antimicrobial activity is one of the pivotal capacities of probiotics to modulate the microbiota. As shown in Figure 5A, the genome of BGI-J9 was found to contain one gene cluster encoding antimicrobial peptides, the core genes *plnE, plnF, plnA, plnK, plnN* and *plnJ* encoded six types of plantaricins (Plantaricin_E, Plantaricin_F, Plantaricin_A, Plantaricin_K, Plantaricin_N and Plantaricin_J). These bacteriocins produced by *L. plantarum* exert antimicrobial effects by disrupting cell membrane integrity. Furthermore, the antimicrobial peptide gene clusters were predicted to contain accessory genes involved in plantaricin biosynthesis, such as *HlyD*, *LanT* and *GlyS*, which facilitate bacteriocin maturation, secretion and transport. Additionally, five secondary metabolite biosynthetic gene clusters were annotated in the BGI-J9 genome (Figure 5B), including four antimicrobial-associated clusters: RiPP-like (unspecified ribosomally synthesized and post-translationally modified peptide product), T3PKS (type III polyketide synthase), Terpene-precursor and Terpene. The organic acid biosynthesis genes (Supplementary Table S2) annotated in BGI-J9, such as *mleS, ldh* and *fab*, also contribute certain antimicrobial activity. *In vitro* experiments have demonstrated that BGI-J9 exhibited significant antimicrobial activity against *S. aureus*, *E. coli*, *P. aeruginosa*, *E. cloacae* and *F. nucleatum*, with inhibition rates ranging from 75.1–92.5% (Figure 5C).

### 3.6 Antioxidant capacity

Overloaded ethanol metabolism leads to a significant accumulation of ROS, causing oxidative stress and inflammatory responses, which in turn induce DNA damage, apoptosis and even tissue damage (Wu and Cederbaum, 2003). KEGG pathway analysis identified 20 antioxidant-related pathways in BGI-J9, involving a total of 44 genes (Table 2). These identified genes in BGI-J9 mainly encoded enzymes related to maintaining redox homeostasis and alleviating oxidative damage, including glutathione peroxidase family, GSH, catalase, thioredoxin and methionine sulfoxide reductase. Additionally, genes encoding antioxidant-associated transport proteins, such as specific ATP-Binding Cassette transporters (ABC transporters) and Energy-Coupling Factor transporter (ECF transporters), were detected, which may further enhance the oxidative stress resistance of BGI-J9. It was further found that BGI-J9 exhibited favorable radical scavenging, with 40.02% scavenging of hydroxyl radicals in particular (Table 3).

**Table 2:** Antioxidant activity-related genes in BGI-J9.

KO number	Genes	Description	EC	**Gene count**
K00432	*gpo*	The glutathione peroxidase family	1.11.1.9	1
K01919	*gshAB*	The glutamate-cysteine ligase type 1 family	6.3.2.2	4
K16786	*gshF*		–	4
K16787	*gshF*		–	4
K00383	*gor*	Glutathione reductase (NADPH)	1.8.1.7	4
K03781	*katA*	Catalase	1.11.1.6	2
K03885	*ndh*	NADH dehydrogenase	1.6.99.3	2
K17869	*nox*	NADH oxidase	1.6.3.4	1
K05910	*npr*	NADH peroxidase	1.11.1.1	2
K06191	*nrdH*	Glutaredoxin	–	1
K11065	*tpx*	Thiol peroxidase	1.11.1.15	1
K03671	*trxA*	Thioredoxin	–	3
K00384	*trxB*	Thioredoxin reductase	1.8.1.9	1
K04083	*hslO*	Molecular chaperone Hsp33, the redox-regulated molecular chaperone prevents irreversible aggregation of thermally unfolding and oxidatively damaged proteins, playing a key role in the bacterial defense system against oxidative stress	3.2.2.23, 4.2.99.18	1
K07304	*msrA*	Peptide-methionine(S)-S-oxide reductase	1.8.4.11	3
K07305	*msrB*	Peptide-methionine(R)-S-oxide reductase	1.8.4.12	1
K08968	*msrC*	L-methionine(R)-S-oxide reductase	1.8.4.14	1
K10563	*fpg*	The enzyme repairs oxidized DNA by removing damaged bases, cleaving the backbone, and creating single-strand breaks	3.2.2.23,4.2.99.18	1
K00158	*poxL*	Pyruvate oxidase	1.2.3.3	5
K21562	*rcfA*	Transcriptional regulator, Crp Fnr family	–	2

**Table 3:** Antioxidant activity of BGI-J9.

DPPH radical Clearance rate/%	ABTS radical Clearance rate/%	Hydroxyl radical Clearance rate/%
10.03 ± 1.07	18.61 ± 0.79	40.02 ± 4.99

### 3.7 Alcohol degrading activity

**Figure 6:**
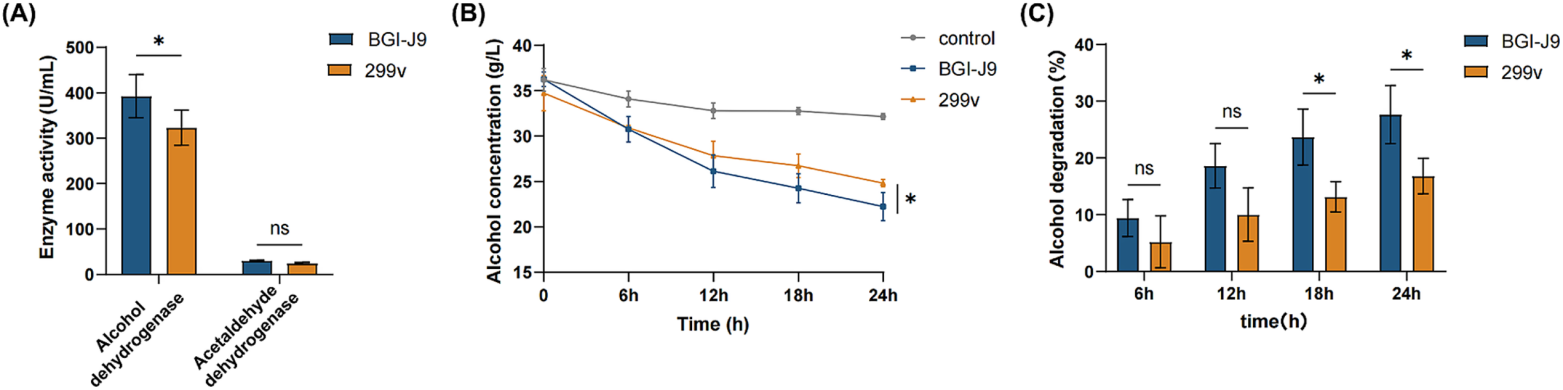
Alcohol degradation capacity of BGI-J9 and 299v. **(A)** ADH and ALDH activities of BGI-J9 and 299v; **(B)** the variation of alcohol concentration and **(C)** degradation rates during the 24-h incubation period.

To further investigate whether BGI-J9 is capable of metabolizing ethanol, genes encoding alcohol degrading enzymes were mined by genome annotation. The results showed that BGI-J9 and 299v share the same number of genes encoding alcohol degradation. BGI-J9 possesses *adh* genes encoding conventional alcohol dehydrogenases for ethanol oxidation, along with *adhC* which encodes a zinc-dependent class III alcohol dehydrogenase that demonstrates high ethanol-binding affinity and dehydrogenation capacity (Table 4). Notably, four *adhE* genes were identified in BGI-J9, encoding bifunctional enzymes with both ADH and ALDH activities. *In vitro* assay revealed that the ADH activity of BGI-J9 was significantly higher than 299v, reaching 392.667 ± 47.35 U/mL, whereas the ALDH activity was comparable (Figure 6A). Moreover, the ethanol metabolism efficiency of BGI-J9 was consistently higher than that of 299v during 24 h, reaching 28.79% (Figures 6B,C). These results indicated that BGI-J9 demonstrated favorable alcohol degrading ability in both genotype and phenotype.

**Table 4:** Alcohol degrading enzyme genes in BGI-J9.

Types of dehydrogenases	KO Number	Genes	Description	EC	**BGI-J9 Gene count**
Catalyzing ethanol oxidation	K00001	*adh*	Alcohol dehydrogenase	1.1.1.1	7
	K00055	*adhC*	Zn-dependent alcohol dehydrogenases, class III	1.1.1.90	1
Catalyzing ethanol and acetaldehyde oxidation	K04072	*adhE*	Acetaldehyde dehydrogenase/alcohol dehydrogenase	1.1.1.1, 1.2.1.10	4

## 4 Discussion

Advanced whole-genome sequencing technology has ushered in a new perspective on probiotic function discovery, accelerating deeper insights into their functional mechanisms. Employing whole-genome sequencing technology, this study obtained a highly complete genome of BGI-J9 and its fundamental characteristics through gene annotation. Concurrently, combined with phenotypic analysis, BGI-J9 was found to show favorable gastrointestinal tolerance, antimicrobial activity and antioxidant activity, as well as notable alcohol-degrading capacity. In brief, this study offered a theoretical foundation for BGI-J9 as a supplement to ameliorate alcohol metabolism and alcohol-induced damage.

The extreme environment of the gastrointestinal tract, including gastric acid, bile salts and digestive enzymes, is a major obstacle limiting the survival and functionality of probiotics. H^+^ in gastric acid alters the membrane potential of probiotic cells, subsequently changing membrane permeability and leading to cell death due to leakage of cellular contents (Halder et al., 2015). In this study, BGI-J9 exhibited robust tolerance to gastric acid, possibly by maintaining intracellular pH through proton efflux. The genes *atpA, atpB, atpC, atpD, atpE, atpF, atpG* and *atpH*, annotated in the genome of BGI-J9, encoded F-type proton-translocating ATPases, which utilize the energy from ATP hydrolysis to actively pump H^+^ out of the cell, reducing intracellular proton accumulation. Among these, the protein encoded by *atpB* functions as a proton sensor, detecting acid stress and activating the downstream acid resistance (AR) gene (Wang and Xu, 2024). Not only that, the *nhaC, nhaP2, nhaP4* and *nhaK* genes encode Na^+^/H^+^ antiporters, which transport Na^+^ into the cell while expelling H^+^, thereby regulating intracellular pH and maintaining ion homeostasis (Pedersen and Counillon, 2019). Furthermore, BGI-J9 might alleviate intracellular H^+^ accumulation through the glutamate-dependent acid resistance system. The *gadB* gene annotated in its genome is a crucial component of this system, encoding glutamate decarboxylase. This enzyme catalyzes the decarboxylation of glutamate while concomitantly consuming intracellular H^+^, thereby mitigating acid stress (Pennacchietti et al., 2018). Bile salts impair cellular membrane integrity, adversely affecting probiotic survival (Urdaneta and Casadesús, 2017). Several genes that help resist bile salt stress have been annotated in BGI-J9. For instance, the product encoded by the *cbh* gene hydrolyzes conjugated bile acids (Leer et al., 1993), thereby enhancing the survival of BGI-J9 in bile salt environments. The proteins encoded by the *ppaC, cfa* and *glnA* genes help maintain intracellular homeostasis and metabolic stability under bile salt stress (Choi et al., 2020; Teixeira and Fidalgo, 2009). The *oppA-D* and *oppF* genes encode an oligopeptide transport system, ensuring nutrient uptake under stress conditions (Lubkowitz, 2011). A series of the *rps* gene encoded ribosomal proteins, supporting normal protein synthesis during bile salt exposure (Zhou et al., 2015). Digestive enzymes degrade proteins and lipids in probiotic cell walls and membranes, compromising cellular integrity. During metabolic processes, the *mleS, ldh, ackA and fab* genes annotated in the BGI-J9 genome produces organic acids such as lactic acid and acetic acid. The accumulation of these acids significantly decreased environmental pH, thereby reducing the activity of digestive enzymes and consequently diminishing their degradation effects on the bacterium. Also, the energy metabolism-related genes (*celA, celB, celC*) enable BGI-J9 to utilize various carbon sources for energy metabolism under stress conditions to maintain basic growth. The identification of these tolerance-related genes indicated that BGI-J9 could employ multiple mechanisms to sustain intracellular homeostasis, nutrient acquisition and metabolic function under extreme conditions, which accounts for the high survival rate of BGI-J9 in the gastrointestinal environment. These characteristics enhanced the intestinal colonization potential of BGI-J9, which is essential for benefiting the host.

Antimicrobial activity is a critical probiotic property of beneficial bacteria and serves as a key manifestation in mitigating alcohol-induced damage. Chronic alcohol intake damages the intestinal barrier and causes gut microbiota dysbiosis, promoting the overgrowth of certain Gram-negative pathogenic bacteria. This leads to increased release of lipopolysaccharide (LPS), which translocates to the liver via the portal vein and subsequently induces liver injury (Wang et al., 2010). Studies have demonstrated that *L. plantarum* J26 reduced the abundance of Gram-negative pathogens in the intestines of mice with alcohol-induced liver injury, preserves intestinal barrier integrity, and alleviates alcohol-induced hepatic inflammation (Li et al., 2022). It is hypothesized that BGI-J9 might similarly mitigate alcohol-induced gut dysbiosis and alleviate inflammation in the intestine and digestive system by suppressing pathogenic microbial proliferation. Encouragingly, in this study, multiple genes associated with antimicrobial activity were annotated in the BGI-J9 genome, including those encoding bacteriocins, secondary metabolites, lactic acid, SCFAs, among others. Genes (*plnA*, *plnE, plnF, plnN, plnK* and *plnJ*) encoding plantaricin were identified in the BGI-J9 genome. It is reported that Plantaricin A, Plantaricin E, Plantaricin F, Plantaricin K and Plantaricin J encoded by the aforementioned genes are monopeptide or dipeptide bacteriocins that interact with cell membranes to increase membrane permeability, causing the leakage of cell contents and ultimately leading to bacterial death (Hauge et al., 1999; Kristiansen et al., 2005; Todorov, 2009). Four antimicrobial-associated secondary metabolite biosynthetic gene clusters: RiPP-like, T3PKS, Terpene-precursor and Terpene, which are capable of synthesizing antibacterial compounds such as RiPPs, polyketides and terpenoids to modulate gut microbiota composition (Guimarães et al., 2019; Li et al., 2024; Zhang et al., 2025). Furthermore, numerous genes encoding organic acid (including lactic acid and acetic acid) production were annotated in the BGI-J9 genome. These organic acids could penetrate pathogenic bacterial cells, interfere with intracellular protein function, and disrupt cellular structures, thereby achieving antimicrobial effects (Kashket, 1987). *In vitro* antibacterial assays confirmed its inhibitory effect on harmful bacteria. These results fully demonstrated the antimicrobial activity of BGI-J9 and its potential to modulate the gut microbiota for alleviating alcohol-induced liver injury. However, further *in vivo* studies are required to validate its efficacy.

Oxidative damage is a major harmful effect of alcohol on the human body, leading to alcoholic fatty liver disease and even progression to liver cancer (Michalak et al., 2021). Chronic alcohol consumption produces excessive ROS, which impairs cellular functions, exacerbates oxidative stress, and ultimately leads to cell death (Tan et al., 2020; Wu and Cederbaum, 2003), while simultaneously depleting intracellular antioxidants like GSH and reducing oxidative stress tolerance (Tan et al., 2020). Previous studies have demonstrated that antioxidant activity is the essential factor of probiotics to mitigate alcohol-induced liver injury (Ren et al., 2024). In this study, numerous genes related to antioxidant activity were annotated in the BGI-J9 genome, implying that BGI-J9 has the potential to alleviate oxidative stress via multiple mechanism. Specifically, the *ndh*-encoded NADH dehydrogenase (a key respiratory chain enzyme) directly scavenges ROS via redox activity while regulating other antioxidant enzymes to enhance cellular defense (Corpas and Barroso, 2014). The *gshAB*, *gshF* and *gshR* genes support glutathione synthesis/reduction, facilitating GSH-dependent ROS clearance. These genes are critical for neutralizing ROS and protecting cells (Silvagno et al., 2020), while also serving as cofactors for antioxidant and detoxification enzymes (Averill-Bates, 2023). Additionally, *katA*-encoded catalase decomposes hydrogen peroxide via iron/manganese cofactors to prevent peroxide-induced damage (Rasheed, 2024), while thioredoxin (encoded by *trxA/trxB*) acts as an electron donor for glutathione peroxidase in peroxide reduction and regulates redox signaling (Ren et al., 2017). Furthermore, *msrA/msrB*-encoded proteins restore oxidized methionine residues to maintain proteostasis (Shcholok and Eftekharpour, 2024). *In vitro* experimental results validated these genomic annotations, confirming that BGI-J9 exhibits excellent antioxidant capacity. The antioxidant capacity of BGI-J9 suggested its potential to ameliorate alcohol-induced oxidative damage.

Alcohol is primarily metabolized in the liver by ADH and ALDH, which sequentially convert ethanol to acetaldehyde and then to acetate (Jiang et al., 2020). Genomic analysis of BGI-J9 revealed seven ADH-encoding genes, demonstrating high catalytic activity for ethanol dehydrogenation. The annotated *adhC* gene encoded a zinc-dependent class III alcohol dehydrogenase that converts ethanol to acetaldehyde while reducing NAD^+^ to NADH, completing the first step of alcohol metabolism. This process also helped maintain intracellular glutathione levels and enhance cellular antioxidant activity (Kidd et al., 2007). Additionally, the BGI-J9 genome contained four *adhE* genes encoding bifunctional enzymes with both alcohol/acetaldehyde dehydrogenase activities, enabling direct conversion of ethanol to acetate (Hitschler et al., 2021). Notably, while multiple ethanol dehydrogenase genes were identified, fewer genes encoding acetaldehyde-oxidizing enzymes were found. This genomic pattern correlated well with phenotypic results showing high ADH activity but relatively low ALDH activity in BGI-J9. 299v is a well-studied probiotic that confers multiple benefits for improving intestinal disorders and health (Ducrotté et al., 2012; Rudzki et al., 2019), but there are few reports on its role in promoting alcohol degradation. Although the same types and quantities of alcohol-degrading enzyme genes as BGI-J9 were annotated in 299v, the ADH activity was significantly lower. This difference might be related to transcriptional levels and post-translational modifications of the genes. Thus, it is not difficult to infer that the enhanced alcohol degradation ability of BGI-J9 is due to the alcohol degradation enzyme genes and their higher expression levels. The *in vitro* alcohol degradation assays, BGI-J9 demonstrated significantly better performance than the commonly used commercial strain 299v. These findings collectively indicated that BGI-J9 possesses active enzymes that directly assist the body in metabolizing alcohol and accelerate its degradation. It is possible to achieve high-efficiency expression of dehydrogenases suitable for functional food applications through genetic engineering techniques in the future. In conclusion, BGI-J9 shows substantial potential in improving alcohol metabolism.

In this study, various probiotic potentials of BGI-J9 were uncovered, since numerous genes associated with gastrointestinal tolerance, antibacterial activity, antioxidant activity and alcohol degradation capacity were annotated, followed by *in vitro* assays to further validate the probiotic properties. These findings collectively demonstrated the potential application as supplements to accelerate alcohol metabolism and reducing liver damage. Notwithstanding, more in-depth animal experiments and clinical trials need to be conducted to further validate the positive effects of BGI-J9 on alcohol metabolism and liver protection. In summary, this study first presented the high-quality complete genome of BGI-J9, laying the scientific foundation for its application in improving alcohol metabolism and liver injury as supplements in the future.

## 5 Conclusion

In conclusion, this study presents the complete genome map of *Lactiplantibacillus plantarum* BGI-J9. Through comprehensive genomic analysis coupled with in vitro experiments, we characterized BGI-J9 as a probiotic strain exhibiting robust gastrointestinal tolerance, antimicrobial activity, and antioxidant capacity. Furthermore, genes associated with alcohol degradation were identified in its genome, and its alcohol degradation-promoting capability was experimentally validated. These findings suggest that BGI-J9 holds promise as a potential probiotic for enhancing alcohol metabolism and alleviating alcohol-induced liver injury.

## Data Availability

The datasets presented in this study can be found in online repositories. The names of the repository/repositories and accession number(s): China National GenBank Database (CNGBdb): CNP0007647 (https://db.cngb.org/search/?q=CNP0007647).

## Footnotes

^1^ https://itol.embl.de/

^2^ https://jspecies.ribohost.com/jspeciesws/

^3^ https://proksee.ca/

^4^ http://eggnog5.embl.de/

^5^ https://www.genome.jp/kegg/

^6^ http://bagel4.molgenrug.nl/

^7^ https://antismash.secondarymetabolites.org/
