# Supplementary material for: Revealing the potent probiotic properties and alcohol degradation capabilities of Lactiplantibacillus plantarum BGI-J9 by combining complete genomic and phenotypic analysis
Source: Front Microbiol. 2025 Sep 9;16:1664033. doi: 10.3389/fmicb.2025.1664033 (PMC12454333; doi:10.3389/fmicb.2025.1664033)
Supplement: Supplementary file 1 [file Table_1.DOCX]

**Table S1** **Carbohydrate-metabolizing enzyme-related genes in BGI-J9.**

| Name | **Gene count** | Description |
| --- | --- | --- |
| Auxiliary Activity redox enzymes (AAs) | 1 | Involved in redox reactions during carbohydrate metabolism |
| Carbohydrate-Binding Modules (CBMs) | 4 | Polysaccharide-binding domain that mediates enzyme-substrate interactions |
| Glycoside Hydrolases (GHs) | 22 | Functions as a component/catalytic element of polysaccharide hydrolases, participating in polysaccharide hydrolysis |
| Glycosyltransferases (GTs) | 27 | Participates in polysaccharide biosynthesis, with some serving as glycosyltransferase subunits |

**Table S2 Organic acid biosynthesis-related genes in BGI-J9.**

| KO number | Genes | Description | EC | **Gene count** |
| --- | --- | --- | --- | --- |
| K00027 | *mleS* | malate dehydrogenase | 1.1.1.38 | 2 |
| K00016 | *ldh* | L-lactate dehydrogenase  D-lactate dehydrogenase  Catalyzes the formation of acetyl phosphate from acetate and ATP. Can also catalyze the reverse reaction | 1.1.1.27 | 16 |
| K03778 | *ldhD* |  | 1.1.1.28 | 2 |
| K00925 | *ackA* |  | 2.7.2.1 | 14 |
| K00645 | *fabD* | Malonyl CoA-acyl carrier protein transacylase | 2.3.1.39 | 6 |
| K09458 | *fabF* | Catalyzes the condensation reaction of fatty acid synthesis | 2.3.1.179 | 3 |
| K00059 | *fabG* | Enoyl-(Acyl carrier protein) reductase | 1.1.1.100 | 8 |
| K00648 | *fabH* | Catalyzes the condensation reaction of fatty acid synthesis | 2.3.1.180 | 4 |
| K00208 | *fabI* | Enoyl- acyl-carrier-protein reductase NADH | 1.3.1.10,1.3.1.9 | 4 |
| K02371 | *fabK* | Nitronate monooxygenase | 1.3.1.9 | 2 |
| K02372 | *fabZ* | Involved in unsaturated fatty acids biosynthesis. | 4.2.1.59 | 3 |
